# Developing and testing an Arduino-based microcurrent stimulator to mimic marine electric pollution on benthos

**DOI:** 10.1016/j.heliyon.2023.e23281

**Published:** 2023-12-06

**Authors:** Davide Lattanzi, Marica Pagliarini, Federica Rebecchi, Fabrizio Frontalini, Patrizia Ambrogini

**Affiliations:** a Department of Biomolecular Sciences, University of Urbino Carlo Bo, 61029 Urbino, Italy; b Department of Pure and Applied Sciences, University of Urbino Carlo Bo, 61029 Urbino, Italy

**Keywords:** Prototype, Low-cost microcurrent device, Biological model, Electric stimulation

## Abstract

The lack of economic funds commonly represents a limiting factor in scientific research and prevents scientists from developing brilliant ideas. Indeed, a new project may involve using appropriate scientific instruments and concurrently dealing with the costs before pursuing new research fields. The innovative concept of investigating the effects of electric fields, as a simulation of marine electrical pollution, on benthic organisms such as foraminifera (marine protozoa) has been recently explored by our research group. This pioneering research has resulted in the development of a cost-effective instrument capable of generating customized electric stimulation patterns with accuracy and reliability. Here, we describe the construction of a low-intensity electrical stimulator based on an Arduino programmable board and a few electronic components. The instrument results very stable and precise regarding the stimulation times and the regulation of the current intensity applied to the biological preparation. Moreover, the setup can stimulate the preparation in constant or pulsed direct current. This homemade stimulation apparatus can be improved or modified according to the researchers’ needs, as possibilities and fields of application can be innumerable.

## 1 Introduction

Controlled or uncontrolled human activities affect the marine environment in several ways such as by introducing harmful substances including plastic, overheating waters, and using sources that generate or transport electric energy [[1], [2], [3]]. Marine pollution by electric fields is undoubtedly the least studied among the pollution sources, and only a few works have been published [[4], [5], [6], [7], [8]]. Therefore, this topic could take on considerable interest in environmental research given the numerous sources of electrical pollution on the seabed, such as the electric fields generated by submarine cables or wind farms.

Benthic foraminifera, marine protozoa, are largely used as pollution bioindicators in marine environments [9] and could represent a good indicator also for electric field pollution. Some benthic foraminiferal species can be easily cultured in artificial seawater by controlling a set of physico-chemical parameters of water, namely temperature, light-dark cycles, and water oxygenation. Recently, *Amphistegina lessonii* was used to test the effects of short-term different electric current densities [10]. To this end, a stimulator capable of generating adjustable direct, constant, or pulsed current stimuli in a range of a few milliampere/microamperes with a potential difference of a few volts and possibly with multiple independent output channels has been developed. Several high-precision stimulators on the market allow for stimulating biological preparations such as NL800A Current Stimulus Isolator (about 600 $), A385 stimulus isolator WPI (about 2000 $), A-*M*-System model 2300 (about 1800 $). These stimulus isolators are designed to deliver precise electrical pulses to biological preparations such as neurons, cardiac cells, and muscle cells to study the electrical properties of cells and tissues [[11], [12], [13]]. These instruments can fine-tune stimulation intensities and duration, but commonly have few output channels. Thus, they must be commonly coupled to timers or computers that set the stimulation timing characteristics. For this reason, the stimulation setup can be difficult to transport and very expensive.

Therefore, taking into consideration our experimental design, as well the costs and dimensions of the stimulators available on the market, we decided to develop a new instrument using the programmable board Arduino Uno or Arduino Nano, which are present on the market at very low prices and are easy to program and connect to external electrical circuits. The present work has been conceived to demonstrate the potential of the Arduino programmable board as a high-precision and versatile stimulator that can effectively stimulate biological preparations such as benthic foraminifera in the range of a few μA per cm^2^. We performed several experiments to assess the precision of the programmable board in generating stimuli and accurately reading currents. Additionally, we guided how to properly adjust the code's delays to achieve stimuli of appropriate duration. Finally, we provided the design of the entire circuit and the code so that other researchers can be easily reproduced it even without an in-depth knowledge of electronics and programming.

## 2 Methods and results

### 2.1 Arduino programable board

Arduino boards are small programmable devices used to build electronic projects at a hobby or professional level. They consist of a microcontroller, the board's brain, and several input/output pins that can be used to connect sensors, actuators, and other components. Arduino boards come in various sizes and shapes, the most common being the Arduino Uno and Arduino Nano. The Arduino Uno has 14 digital input/output pins, six analog inputs, a 16 MHz quartz crystal, a USB connection, and a power jack. The Arduino Nano is a smaller version of the Uno, with 22 digital input/output pins, eight analog inputs, and a smaller footprint. Both boards can be programmed using the Arduino Integrated Development Environment (IDE), a software tool that allows us to write, compile, and upload code to the board. The IDE uses a simplified version of the C++ programming language and has several built-in libraries and examples that make it easy to start. The digital input/output pins on Arduino boards are designed to handle a maximum current of 40 mA and a maximum voltage of 5 V. The analog input pins allow the board to read analog voltage values from external sensors or devices. These analog channels convert the input voltage to a digital value using an analog-to-digital converter (ADC) built into the microcontroller. The ADC resolution, or the number of bits used to represent the analog value, is normally 10 bits, resulting in a range of 0–1023 for the converted digital values. On an Arduino Uno and Arduino Nano, this yields a resolution between readings of 5 V/1023 units or 0.0048 V (4.8 mV) per unit. As digital I/O pins, the analog input pins on Arduino boards have a limited voltage range, commonly between 0 and 5 V.

Based on the size of the foraminiferal containers and the electrodes' resistance, our calculations indicated that the digital outputs could produce sufficient current to generate an electric field with appropriate current density, eliminating the need for external power supplies and more complex circuit design. Finally, the analog channels can be used with a sensing resistor to independently monitor the current delivered to the output channels. We selected Arduino Nano as the optimal choice for constructing our instrument due to its ability to accommodate eight analog inputs and measure the current across four distinct stimulation channels.

### 2.2 Description of the circuit for foraminiferal stimulation

We employed an Arduino Nano open-source electronic prototyping board (Arduino Italy) with an ATmega328 microcontroller. The board was programmed using the Arduino open-source IDE to generate low-intensity current stimulation, either constant or pulsed, directly from the digital channels and to measure the current stimulation intensity that flows through the electrodes using analog input channels. The Arduino board was connected to a Liquid Crystal Display (LCD) 16X2 to display the current stimulus intensity.

**Fig. 1:**
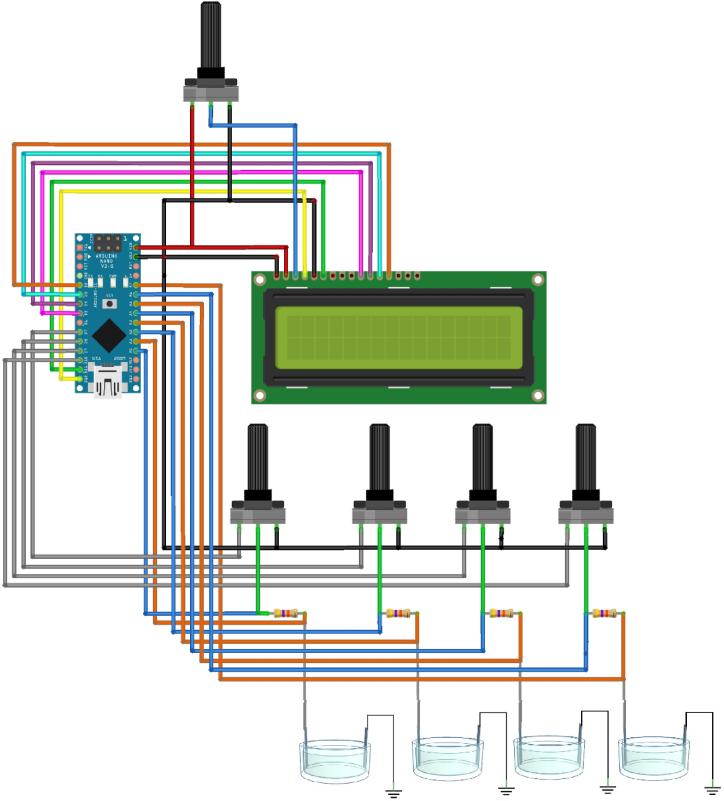
Summary diagram of stimulator architecture.

The rectangular-flat electrodes were made up of platinum (dimensions: 4 mm in width, 0.2 mm in thickness) or stainless steel (dimension: 90 mm in width, 0.2 mm in thickness) and were placed in a multiwell plate (3.5 cm diameter, i.e., UltraCruz® Tissue Culture six wells sterile plate) each filled with 9.6 ml of artificial seawater and/or in a rectangular container (140 mm × 93 mm) filled with 150 ml of artificial seawater, respectively. Both types of the electrode were immersed for about 1 cm (Fig. 1). The artificial seawater was prepared in accordance with the composition indicated in ASTM D1141- 98 [14]. The electrical resistance for the platinum electrodes in the multiwell plate reached about 3 kOhm when they were dipped in artificial seawater. At the same time, the stainless-steel electrodes immersed with the same solution in the large container reached about 2.5 kOhm. Each Arduino Nano board had the capability to control four pairs of electrodes. The positive platinum electrodes (anodes) were connected to the chip pins D8, D9, D10, and D11 via 50K potentiometers in series with 47K resistors, specifically chosen to achieve a current range up to 100 μA. To measure the current flow, the chip pins A1-A7 were connected in pairs across the 47K resistors (used as sensing resistor).

**Fig. 2:**
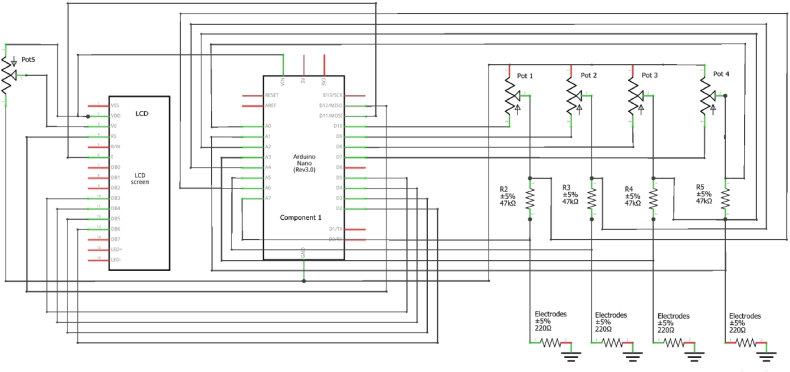
Stimulator wiring diagram.

The 50K potentiometers were utilized to adjust the current intensity as necessary. Finally, the negative electrodes (cathodes) were connected to the Arduino ground. We measured the current flowing in each stimulation channel using the analog channels and a known resistance (sensing resistor). A sensing resistor is a type of resistor designed to measure the current flowing through a circuit. It works by producing a voltage drop proportional to the current flowing through it, according to Ohm's law (V=IR), where V is the voltage, I is the current, and R is the resistance of the sensing resistor. The voltage drop across the sensing resistor is commonly very small, typically in the millivolt range. The value of the sensing resistor is carefully selected to ensure that it does not significantly affect the circuit's overall performance or voltage drop. In our case, we decided to use a sensing resistor with a high value (47K) for two main reasons: i) to increase the resistance of the circuit to limit the maximum current that can be supplied; ii) to create a potential drop readable from the Arduino analog channel without using voltage amplifiers. Table 1 provides a detailed list of parts and Fig. 2 shows the wiring diagram.

**Table 1:** Part list of Arduino-based stimulator.

Part list		
Label	Part Type	Properties
Component 1	Arduino Nano (Rev3.0)	Arduino Nano (3.0)
Electrodes		Platinum or Stainless steel
LCD	LCD screen	16X2 character
Pot 1	Potentiometer 50 KΩ	Linear
Pot 2	Potentiometer 50 KΩ	Linear
Pot 3	Potentiometer 50 KΩ	Linear
Pot 4	Potentiometer 50 KΩ	Linear
Pot 5	Potentiometer 10 KΩ	Linear
R2	47 kΩ Resistor	¼ Watt
R3	47 kΩ Resistor	¼ Watt
R4	47 kΩ Resistor	¼ Watt
R5	47 kΩ Resistor	¼ Watt

### 2.3 Arduino code for pulsed direct current stimulation

**Fig. 3:**
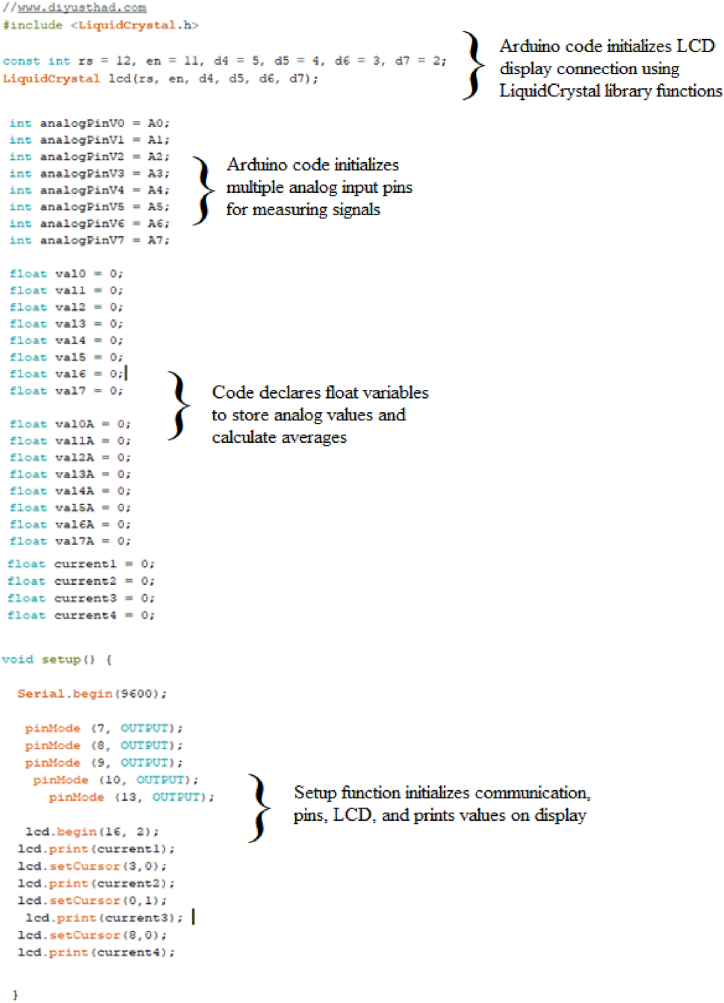

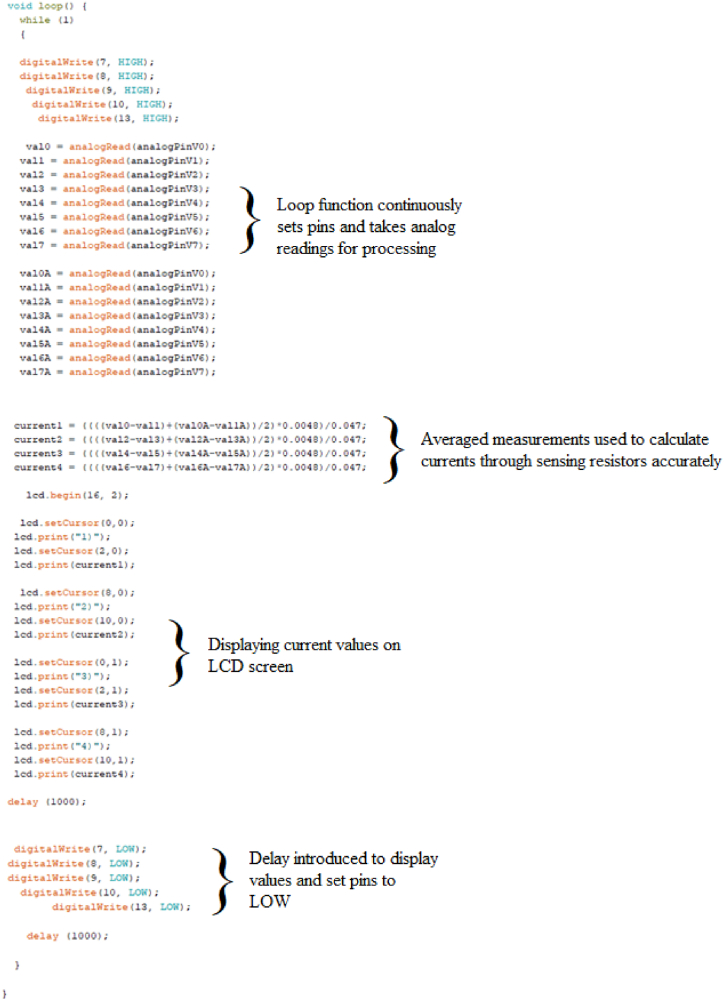
Arduino code with description.

A detailed description of the sketch of the code for obtaining pulsed microstimulation in direct current is provided in order to enhance the comprehensibility of the utilized Arduino code (Fig. 3).

The code begins with the inclusion of the LiquidCrystal library, which allows the Arduino to communicate with the 16x2 LCD display using a set of predefined functions. Next, the sketch defines the pins that are used to connect the LCD display to the Arduino. These are assigned to the variables "rs", "en", "d4", "d5", "d6", and "d7", with their corresponding pin numbers specified. Finally, the "LiquidCrystal" object is created using the previously defined pins, allowing the sketch to use the library functions to write characters and messages to the LCD display. Overall, this code initializes the connection between the Arduino and the LCD display, enabling it to display information as programmed.

The lines of code described above initialize multiple analog input pins on the Arduino board to measure analog signals. The pins are declared using the "int" keyword and assigned to the variables analogPinV0 through analogPinV7, with each variable containing a different PIN corresponding to a specific analog input pin on the Arduino board. The code also declares sixteen float variables, val0 through val7A, which will be used to store the measured analog values from each of the eight input pins. These variables are initialized to 0 at the start of the program. Finally, another section initializes four more float variables (current1 to current4) to 0. These last four variables will be used later in the code to calculate the average values read from the analog channels.

The “void setup ” code segment represents the setup function in Arduino, which is called only once at the beginning of the program. The function starts by initializing the communication between the Arduino board and the computer by calling the Serial.begin function and setting the communication rate to 9600 bits per second. The following lines set up the digital output pins 7, 8, 9, 10, and 13 for controlling four pairs of electrodes and one diode led, respectively. The code then initializes an LCD object and specifies the dimensions of the display (16 columns x 2 rows) using the lcd.begin function. The values of the four current variables are printed on the LCD using the lcd.print function and the lcd.setCursor function is used to set the cursor position for each value.

The next part of the code defines the void loop function. Within this function is an infinite while loop, indicated by the condition while (1). This construct ensures that the code inside the loop will be executed continuously without termination. Inside the loop, digital pins (7, 8, 9, 10, and 13) are set to a high state using the digitalWrite function. This action configures these pins to output a high voltage level (5 V), indicating an active state. Following the digital writes, analog readings are taken from eight analog pins (analogPinV0 to analogPinV7) using the analogRead function. Each reading is assigned to a corresponding variable (val0 to val7) for further processing. The same analog readings are duplicated and stored in variables suffixed with "A" (val0A to val7A). These replicated measurements allow us to average the values by decreasing the variability of the readings. Subsequently, calculations are performed to determine the current values (current1 to current4) based on the analog readings using the following formula: current1 = ((((val0-val1)+(val0A-val1A))/2)*0.0048)/0.047. In this formula, we took a double analog reading across the sensing resistors to calculate the current flowing through each pair of electrodes. The value thus obtained was multiplied by 0.0048 to obtain the correct value in volts. The current was finally calculated through Ohm's law by dividing the calculated potential value by the value of the sensing resistor (47K).

After calculating the current values, the LCD screen is initialized with the lcd.begin (16, 2) code. The current values are displayed on the screen in 4 distinct locations using the lcd.setCursor and lcd.print code. A delay of 1000 ms (1 s) is introduced using the delay function. This pause allows the values to remain displayed on the screen and the output channels to remain at a high logic level (5 V). Finally, the digital pins 7, 8, 9, 10, and 13 are set to a LOW state using the digitalWrite function. Another delay of 1000 ms is introduced, maintaining the digital pins in the LOW state for the specified duration. Acting on the delay values, the stimuli's duration and frequency can be easily changed. Finally, since we want to stimulate the biological preparation with a constant direct current, we must eliminate the section of code assigned to define the initial delay and the portion that sets the digital output channels to a low logic state. However, it is necessary to maintain the last delay to ensure an accurate representation of the values on the LCD screen.

## 3 Test on the accuracy of the stimulation setup

**Fig. 4:**
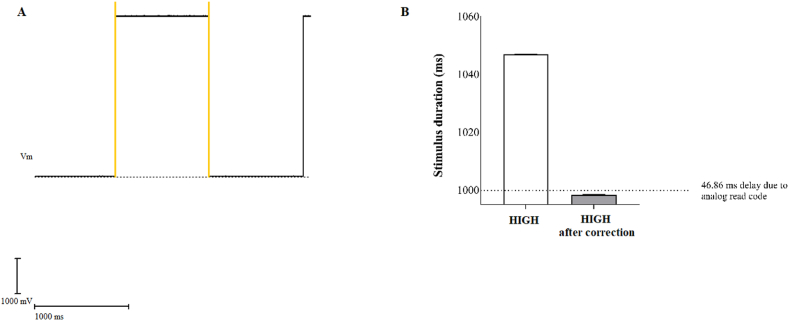
A) Analysis method of the HIGH stimulation duration using WinWCP. The yellow lines indicate the cursor positions for evaluating the length of the stimulation). B) Average duration of the stimulation before and after the code correction, values are expressed as mean ± SD. (For interpretation of the references to colour in this figure legend, the reader is referred to the Web version of this article.)

Our experiments aimed to validate the accuracy of the stimulation output obtained from the given sketch to Arduino. To assess the accuracy of the data, we employed a setup for electrophysiological recordings, which consisted of a PC, a PCI 6221 acquisition board, a BNC 2090A connector, and WinWCP software (John Dempster, University of Strathclyde, UK). The WinWCP software enabled us to record the Arduino output and analyze the signals with high precision in terms of both time and amplitude. Initially, we focused on validating the accuracy of the stimulation duration. To perform this evaluation, we provided a square wave with a duration of 1 s by incorporating 1000 ms delay in the Arduino code after setting the digital channels to the high state and evaluated the period of time in which Arduino digital channels remained in the HIGH state (Fig. 4A). Several evaluations were conducted, and the stimulation duration was found to be 46.84 ms longer due to the time required for code reading. Therefore, we made the necessary code correction considering this delay, and as shown in Fig. 4B, the stimulation duration was perfectly adjusted to 1000 ms.

**Fig. 5:**
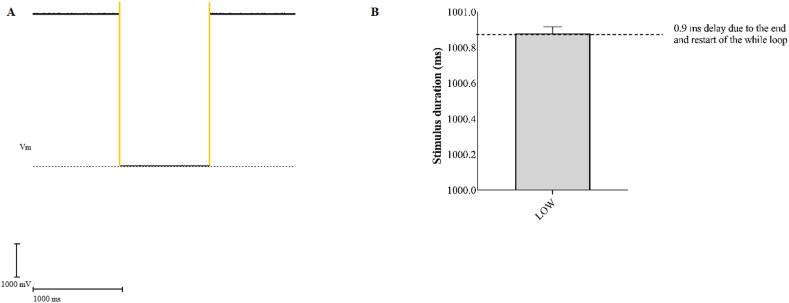
A) Analysis method of the LOW stimulation duration using WinWCP. The yellow lines indicate the cursor positions for evaluating the length of the stimulation. B) Average duration of the LOW state, the values are expressed as mean ± SD. (For interpretation of the references to colour in this figure legend, the reader is referred to the Web version of this article.)

Subsequently, we conducted the same analysis to evaluate whether the duration of Arduino staying in the LOW state corresponded to that set in the sketch. We set a duration of 1 s and, quantified it using WinWCP (Fig. 5A). As shown in Fig. 5B, the duration of the low state of the digital channels was 0.9 ms longer than the delay set in the Arduino sketch, this short period of time is necessary for initiating a while loop cycle.

**Fig. 6:**
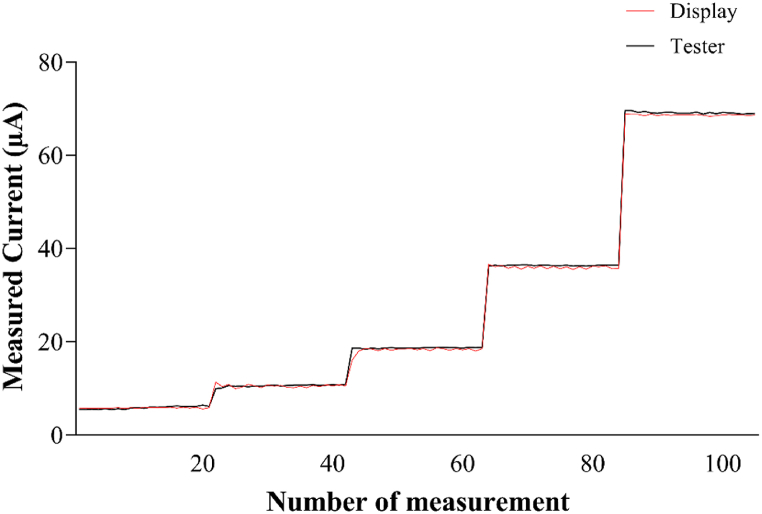
Correspondence between the values displayed on the stimulator's screen and those measured by the tester.

Finally, we evaluated the correspondence between the effective administered microcurrents and the value displayed on the LCD. To perform this, a large container (9.4 × 14.4 cm) containing artificial seawater (prepared in accordance with the composition indicated in ASTM D1141-98) and stainless-steel electrodes immersed to a depth of about 1 cm were used. Five current values (68 μA, 36 μA, 18 μA, 10 μA, 6 μA) were set, and the values displayed on the screen were compared to those detected by a commercially available tester. Twenty measurements were taken for each value recorded by the tester and those shown on the display overlap, confirming the accuracy of the provided current intensity (Fig. 6).

## 4 Discussion

The main goal of this work was to provide information on how to develop a high-precision, low-cost instrument suitable for low-intensity direct current stimulation of vital biological models.

In recent years, there has been a growing trend among researchers to utilize Arduino in the construction of scientific instruments due to its precision and ease of programming [[15], [16], [17], [18], [19]]. Accordingly, we constructed a low-cost stimulator using the programmable Arduino Uno or Arduino Nano board, which empowers researchers to easily modify the temporal characteristics of electrical stimulation. This adaptability enables the assessment of different stimulation protocols on biological preparations. Here, we assessed the precision of the Arduino Nano-based stimulator and provided a comprehensive guide on how to properly set the delays within the code to minimize additional delays caused by code execution. Additionally, we meticulously described the circuitry and each component of the code, aiming to enable researchers with limited knowledge of electronics and programming to replicate and utilize this stimulator effectively.

We tested our self-built device on foraminifera to simulate the effects of electric field pollution on benthic organisms. Foraminifera play a critical role in marine ecosystems [19] and studying their response to electric field pollution offers valuable insights into the potential ecological implications of this type of contamination. By precisely controlling the intensity and duration of exposure, we were able to replicate the conditions that these organisms encounter in areas where electric field pollution is prevalent. Using our homemade stimulator on foraminifera, we observed a dose-dependent negative impact of electrical stimulation on these organisms. Continuous electrical currents exceeding 0.29 μA/cm^2^ led to a marked reduction on pseudopodial activity [10]. However, higher current values were required to produce comparable adverse effects on pseudopodial activity when the stimulation was pulsed [10]. These findings highlight the importance of considering both the intensity and pulsation pattern of electrical stimulation when assessing its impact on foraminifera and other marine organisms. By establishing thresholds at which detrimental effects occur, we can better evaluate the potential risks associated with varying levels of electric field pollution and develop effective mitigation strategies to protect benthic organisms and their ecosystems.

Although there are instruments commercially available that can fulfill the same functions, our instrument has undeniable advantages, such as i) low cost, ii) compact size allowing placement inside incubators, iii) the possibility to conduct extended stimulations due to the independence from batteries, the independence from external digital stimulators or PC, iv) a high number of independent channels enabling various stimulation conditions simultaneously, and v) flexibility in using different protocols by modifying the Arduino code (see Table 2). On the other hand, our instrument has some limitations concerning the need to manually adjust the current in prolonged stimulation protocols due to changes in electrode resistance induced by the accumulation of charges (electrical double layer), and the inability to exceed 40 mA of current amplitude without the use of additional components such as power MOSFETs and external power circuits.

**Table 2:** Comparison between the self-built instrument and the commercially available instruments.

	Stimulation Channels	PC connection	Range	Power supply	Cost
A385 WPI	1	Yes	0–100 mA	Lead Batteries rechargeable	2000 $
NL800A	1	Yes	0–10 mA	Batteries GP23A	600 $
A-M system model 2300	1	Yes	0–10 mA	Integrated Rechargeable battery	1800 $
Low-cost Arduino stimulator	4	No, generates stimulation protocols based on Arduino code	Variable from 0 to 40 mA based on used resistors	Different power supply: Batteries, 5V USB charger, USB PC port	40 $

In conclusion, our homemade instrument has been proven to be highly versatile, easy to build, and has the potential to be used on different biological preparations, such as cultured cells, where stimulation with current intensities in the range of a few microamperes is required.

## Funding

This research did not receive any specific grant from funding agencies in the public, commercial, or not-for-profit sectors.

## Data availability

Data will be made available on request.

## Additional information

No additional information is available for this paper.

## CRediT authorship contribution statement

**Davide Lattanzi:** Writing - original draft, Methodology, Data curation, Conceptualization. **Marica Pagliarini:** Writing - original draft, Methodology, Data curation, Conceptualization. **Federica Rebecchi:** Methodology, Data curation. **Fabrizio Frontalini:** Writing - review & editing, Supervision. **Patrizia Ambrogini:** Writing - review & editing, Supervision.

## Declaration of competing interest

The authors declare that they have no known competing financial interests or personal relationships that could have appeared to influence the work reported in this paper.
